# Reappraising plastid markers of the red algae for phylogenetic community ecology in the genomic era

**DOI:** 10.1002/ece3.5984

**Published:** 2020-01-11

**Authors:** Shing Hei Zhan, Chun‐Chi Shih, Shao‐Lun Liu

**Affiliations:** ^1^ Department of Zoology & Biodiversity Research Centre the University of British Columbia Vancouver BC Canada; ^2^ Department of Life Science & Center for Ecology and Environment Tunghai University Taichung Taiwan

**Keywords:** environmental DNA metabarcoding, phylogenetic inference, phylogenomics, plastid genomes, *rbc*L, *rpoC1*

## Abstract

Selection of appropriate genetic markers to quantify phylogenetic diversity is crucial for community ecology studies. Yet, systematic evaluation of marker genes for this purpose is scarcely done. Recently, the combined effort of phycologists has produced a rich plastid genome resource with taxonomic representation spanning all of the major lineages of the red algae (Rhodophyta). In this proof‐of‐concept study, we leveraged this resource by developing and applying a phylogenomic strategy to seek candidate plastid markers suitable for phylogenetic community analysis. We ranked the core genes of 107 published plastid genomes based on various sequence‐derived properties and their tree distance to plastid genome phylogenies. The resulting ranking revealed that the most widely used marker, *rbc*L, is not necessarily the optimal marker, while other promising markers might have been overlooked. We designed and tested PCR primers for several candidate marker genes, and successfully amplified one of them, *rpoC1*, in a taxonomically broad set of red algal specimens. We suggest that our general marker identification methodology and the *rpoC1* primers will be useful to the phycological community for investigating the biodiversity and community ecology of the red algae.

## Graphical abstract

Environmental DNA metabarcoding utilizes a single genetic marker to determine the species composition of communities and to measure the phylogenetic diversity within and among communities. In a re‐analysis of 107 plastid genomes of the red algae (Rhodophyta), we found that traditional phylogenetic markers, such as *rbc*L and *psbA*, may not necessarily be optimal for those purposes in those organisms. Instead, we uncovered potentially superior candidate markers and proposed and successfully tested a novel marker, *rpoC1*, for future community ecology studies of the red algae

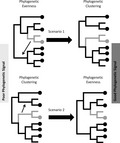

## 1 INTRODUCTION

Integration of phylogenetic information into community ecology has enjoyed an upsurge of interest in the past decade (e.g., Cavender‐Bares, Kozak, Fine, & Kembel, 2009; Webb, Ackerly, McPeek, & Donoghue, 2002; Weber, Wagner, Best, Harmon, & Matthews, 2017). With this marriage of phylogenetics and ecology, we can better explore the processes shaping biodiversity and driving community assembly in an evolutionary context. The recent introduction of *environmental* DNA (eDNA) metabarcoding (i.e., identification of all species in an environmental sample via DNA sequencing) facilitates monitoring of community biodiversity of various organisms in virtually unlimited types of ecological niches. eDNA metabarcoding has been made widely accessible by high‐throughput next‐generation sequencing (HTS), by which millions of pieces of eDNA are sequenced in a massively parallel and cost‐effective fashion. Commonly, eDNA metabarcoding employs a single genetic marker that enables species identification, and the marker can be enriched via PCR amplification (reviewed in Deiner et al., 2017) or target hybridization (i.e., using molecular “baits”; e.g., Wilcox et al., 2018). HTS‐based eDNA metabarcoding has been applied in community ecology (reviewed in Porter & Hajibabaei, 2018), for example, to investigate species turnover in a community (e.g., Hugerth & Andersson, 2017; Pérez‐Valera, Goberna, & Verdú, 2015) and to inform environmental management and conservation efforts (e.g., Brooks et al., 2015; Kress et al., 2009).

Most eDNA metabarcoding studies employ well‐established genetic markers for pragmatic and historical reasons. In practice, a suitable genetic marker is amenable to primer design so as to maximize its PCR amplification efficacy across a variety of species within a group of interest. Considerations include (a) the length of the genetic region to be amplified (typically, it is easier to achieve good amplification for regions less than 1,000 base pairs long) and (b) an appropriate level of nucleotide conservation across the group (i.e., the marker gene should be conserved enough for efficient PCR amplification, and yet it should evolve fast enough for species differentiation; reviewed in Deiner et al., 2017). For animals, plants, and bacteria, there are established DNA barcode genes for biodiversity surveys and community ecology (e.g., *cox1*, *rbc*L, and 16S rRNA). These marker genes are also the cornerstone of molecular systematics and phylogenetics (e.g., Freshwater, Fredericq, Butler, Hommersand, & Chase, 1994; Lahaye et al., 2008; Smith, Woodley, Janzen, Hallwachs, & Henert, 2006). Thus, for those popular markers, large and high‐quality reference databases exist (e.g., Barcode of Life Data System; Ratnasingham & Hebert, 2007).

**Figure 1:**
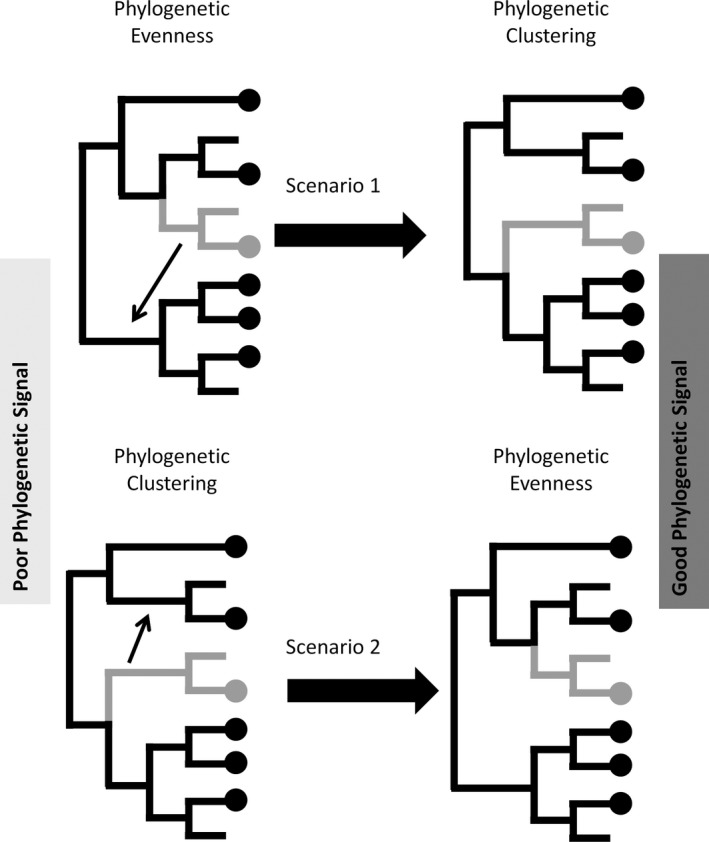
Schematic illustrating how phylogenetic misplacement of a taxon (gray dot) may inflate the phylogenetic diversity of an ecological community (Scenario 1) or deflate it (Scenario 2). Dots at the terminal tips of the inferred phylogeny indicate taxa that are present within a community. Arrows indicate the correct phylogenetic positions of lineages

In phylogenetic community ecology, two important quantities to estimate are relatedness among species *within* a community (i.e., phylogenetic *alpha* diversity) and relatedness among species *between* communities (i.e., phylogenetic *beta* diversity). The measurement of alpha and beta diversity indices can inform us whether or not a given community has greater phylogenetic diversity or more distinct phylogenetic components than other communities (e.g., Daru, Elliott, Park, & Davies, 2017; Kembel et al., 2010). Poor phylogenetic signal, however, may lead to erroneous inferences about phylogenetic relatedness among species within a community or among communities. For instance, considering alpha diversity, phylogenetic misplacement of taxa based on a marker with poor phylogenetic signals, might misleadingly inflate the phylogenetic diversity of a community (e.g., increasing phylogenetic evenness; see Scenario 1 in Figure 1) or deflate it (e.g., increasing phylogenetic clustering; see Scenario 2 in Figure 1). Thus, careful selection of an appropriate marker may be crucial to phylogenetic community analysis.

Traditional organellar marker genes, such as plastid genes, have improved our understanding of biodiversity and community ecology (e.g., Heise, Babik, Kubisz, & Kajtoch, 2015; Porter, Shokralla, Baird, Golding, & Hajibabaei, 2016). For many underexplored groups of eukaryotes (such as algae), it is unclear whether or not widely used markers (e.g., *rbc*L) are the “optimal” choice for phylogenetic community analysis. In the red algae, the commonly used plastid markers—*psaA* (photosystem I P700 chlorophyll a apoprotein A1), *psaB* (photosystem I P700 chlorophyll a apoprotein A2), *psbA* (photosystem II protein D1 2), and *rbc*L (ribulose bisphosphate carboxylase large chain)—individually approximate the red algal tree of life poorly (e.g., Verbruggen et al., 2010). To resolve deep relationships across the red algal phylogeny, multi‐locus and whole plastid genome approaches have been taken (e.g., Boo et al., 2016; Díaz‐Tapia, Maggs, West, & Verbruggen, 2017; Lam, Verbruggen, Saunders, & Vis, 2016; Nelson et al., 2015). In phylogenetic community studies involving eDNA metabarcoding, a single well‐selected locus can still be useful if it can approximate the red algal phylogeny, especially at shallow nodes (i.e., the species‐ or population‐level). As mentioned previously, the reasons to choose one marker over the others have been pragmatic (e.g., the ease of PCR amplification and the availability of a rich sequence database) and grounded on its limited evaluation in focal taxonomic groups (e.g., *psbA* in the reef‐building coralline algae; Broom et al., 2008). The phylogenetic utility of alternative plastid genes—such as the *rpo* (DNA‐dependent RNA polymerase) genes (*rpoA*, *rpoB*, *rpoC1*, and *rpoC2*)—has been explored in several studies of cyanobacteria and land plants (e.g., Palenik & Swift, 1996; CBOL Plant Working Group, 2009; Gomolińska, Szczecińska, Sawick, Krawczyk, & Szkudlarz, 2017). Although it remains to be seen whether or not the *rpo* genes are better phylogenetic markers than other plastid genes, the *rpo* genes have often been selected to be potential complementary markers for the phylogenetic analyses in cyanobacteria and land plants due to their rapid rate of molecular evolution and their PCR amplification efficiency across different major lineages. In the red algae, other plastid genes have seldom been evaluated for biodiversity surveys and phylogenetic community analysis. To the best of our knowledge, only the phycoerythrin gene has been recently proposed by Yang and Boo (2006) for the biodiversity survey of the order Ceramiales. We believe that there may be promising, overlooked plastid genes which are beneficial for investigating the biodiversity and community ecology of the red algae.

Recently, many complete plastid genomes that taxonomically span all the major lineages of the Rhodophyta have been published. Phylogenetic analyses of these genomes have yielded robust species trees of the red algae (e.g., Costa, Lin, Macaya, Fernández‐García, & Verbruggen, 2016; Díaz‐Tapia et al., 2017; Janouškovec, Liu, Martone, Collén, & Keeling, 2013). These plastid genomes form a good foundational resource for analyses requiring an adequate phylogenetic framework. Our group was the first to publish an HTS‐based eDNA metabarcoding study of the red algae (Hsieh et al., 2018); related works performed DNA barcoding in coralline algae, but they did not sequence *environmental* DNA (Bittner et al., 2010; Carro, Lopez, Peña, Bárbara, & Barreiro, 2014). In our previous work which surveyed the biodiversity of cyanidia—a group of unicellular thermoacidophilic red algae (Hsieh et al., 2018; Hsieh, Zhan, Lin, Tang, & Liu, 2015)—we chose *rbc*L because of its PCR amplification efficiency, its single‐copy nature, and the existence of a well‐populated sequence database (with hundreds of entries deposited in NCBI GenBank). While *rbc*L is a powerful tool for eDNA metabarcoding, it is unknown whether or not superior markers may exist for phylogenetic community analysis (to measure phylogenetic alpha and beta diversities). To fill this gap, foundational work is needed that (a) identifies and evaluates candidate markers, (b) designs and tests new PCR primers, and (c) constructs a well‐annotated database for the most promising candidate markers. The growing genomic resource collectively produced by the phycological community presents an unprecedented opportunity to take the first step toward building that foundation—that is, finding superior phylogenetic markers and creating resources to support their usage for biodiversity surveys and community ecology.

In this study, we provided a proof‐of‐concept work to leverage 107 reported red algal plastid genomes to scan for candidate plastid markers that fit our criteria. Using the idea of phylogenetic topological similarity, we devised a simple ranking strategy that involves the comparison of individual plastid gene trees to a single target phylogeny—here, the plastid genome species tree inferred using all core plastid genes. More specifically, we applied normalized Robinson–Foulds distance, a notion of tree distance that measures the proportion of bipartitions unique to one of the two given phylogenetic trees (Robinson & Foulds, 1981); in our study, the greater the distance (i.e., closer to 1), the more the disagreement there is in pairwise tree comparisons, and the more poorly a gene tree approximates the target plastid genome tree. This phylogenomics approach allowed us to assess the commonly used markers (e.g., *psaA*, *psaB*, *psbA*, and *rbc*L) in red algal studies (reviewed in Brodie & Lewis, 2007; Leliaert et al., 2014; Saunders & Moore, 2013), as well as less‐studied markers, to identify better candidates for biodiversity surveys and phylogenetic community ecology.

## 2 MATERIALS AND METHODS

### 2.1 Sequence data collection and processing

We collected 107 publicly available plastid genomes from red algal taxa deposited in NCBI GenBank (Table S1 in Dryad; collected up till Dec. 2017). The sampled taxa represent most of the major orders and families of the Rhodophyta. Using the gene annotations of the NCBI GenBank entries, we extracted all of the protein‐coding sequences and assembled them into 120 single‐copy core gene families represented by at least 96 taxa (i.e., ~90% of the 107 taxa). In a few taxa, we removed genes that had multiple fragmented coding frames (i.e., poor coding sequencing annotations), because they might be genome assembly artifacts and/or incorrect annotations. Also, we excluded one gene (*ccs1*), because it is duplicated across many taxa and paralogs are not ideal markers (for example, see the genus *Polysiphonia*). Next, we translated coding sequences into amino acid (AA) sequences using TransDecoder 3.0.0 (Haas et al., 2013), retaining the longest open reading frame with a minimum AA length of 50. We then aligned the AA sequences using MUSCLE 3.8.31 (Edgar, 2004). Additionally, we obtained the corresponding alignments of the nucleotide (NT) sequences by back translating AAs to their original codons. This processing resulted in AA and NT alignments of 120 gene families, each of which includes up to 107 taxa. This procedure was implemented in Python using the sequence processing functionalities in BioPython 1.70 (Cock et al., 2009). The analysis scripts as well as the data and result files were deposited and archived in the GitHub repository: https://github.com/szhan/rhododb.

### 2.2 Partitioning analysis

Using PartitionFinder2 2.1.1 (Lanfear, Frandsen, Wright, Senfeld, & Calcott, 2016) in conjunction with RAxML 8.2.11 (Stamatakis, 2014), we determined AA and NT data partition groupings (which possess similar substitution models and model parameters) under the r‐clustering algorithm (Lanfear, Calcott, Kainer, Mayer, & Stamatakis, 2014). We identified the best‐fitting AA and NT models for each gene family under the corrected Akaike Information Criterion (Burnham & Anderson, 2002). Under the partition schemes and the associated substitution models, we inferred AA and NT plastid genome species trees and individual gene trees.

First, we inferred two plastid genome species trees (i.e., AA and NT trees), beginning with an AA tree. The AA alignments were partitioned by gene and then grouped using PartitionFinder2. All the AA models implemented in RAxML, including their + G variants, were considered. PartitionFinder2 found 77 AA partition groupings. Under this partition grouping scheme, RAxML was run using the best‐fitting AA models. Next, we obtained a NT plastid genome tree using a similar approach. The NT alignments were partitioned according to the gene‐by‐codon scheme (“G × C”), which treats the first, second, and third codon sites of each NT alignment as separate partitions to be grouped. Thus, the NT substitution models GTR and GTR + G were fitted. This resulted in 282 NT partition groups, and GTR + G was the best model for all the partition groups. RAxML was run on the full NT alignment under the best partition grouping scheme.

Second, with the plastid genome phylogenies in hand, we reconstructed the trees of the individual genes. We estimated two trees for each gene family, one based on its AA alignment and the other based on its NT alignment. The best‐fitting AA and NT models identified during inference of the plastid genome trees were also used to derive the gene trees.

All of the RAxML analyses were performed with 100 rounds of rapid bootstrapping. Also, in all of the phylogenies, we treated Cyanidiophyceae as the outgroup of the remaining taxa, as have other workers (e.g., Yoon, Muller, Sheath, Ott, & Bhattacharya, 2006).

### 2.3 Phylogenetic tree comparisons

To rank the individual plastid genes, we computed the normalized Robinson–Foulds distance (nRF) between each of the plastid gene trees and a target plastid genome tree. Before calculating the distance between a gene tree and a target tree, taxa absent in the gene tree but present in the target tree were pruned from the target tree, and the trees were unrooted. We performed two sets of nRF distance calculations to compare the following: (a) the AA gene trees and the AA plastid genome tree and (b) the NT gene trees and the NT plastid genome tree. For tree processing and nRF distance calculations, we used the R packages *ape* 5.1 (Paradise, Claude, & Strimmer, 2004) and *phangorn* 2.4.0 (Schliep, 2011). Visual juxtaposition of phylogenetic trees was performed with the aid of the R package *phytools* version 0.6‐44 (Revell, 2012).

### 2.4 Estimation of degrees of sequence variation and rates of molecular evolution

For each plastid gene family, we computed its pairwise p‐distance (percentage nucleotide mismatches, which is a simple measure of sequence divergence) using a custom Python script. We also estimated its pairwise rate of nonsynonymous substitution (dN) and its pairwise rate of synonymous substitution (dS) using CodeML (PAML 4.9h; Yang, 2007), taking the median across all the sequence pairs. Lastly, we calculated the proportion of parsimony informative sites using AMAS (Borowiec, 2016). The statistical analyses (regression analysis and correlation tests) were conducted using R (R Core Team, 2018).

### 2.5 PCR experiments and Sanger sequencing

To examine the efficacy of the designed primers on a wide taxonomic spectrum of the Rhodophyta, we selected eleven species that span five different classes: two in Cyanidiophyceae, one in Porphyrideophyceae, one in Compsopogonophyceae, one in Bangiophyceae, and six in Florideophyceae (Appendix 1). The six species in Florideophyceae cover four subclasses: one in Hildenbrandiphycidae, one in Nemaliophycidae, one in Corallinophycidae, and three in Rhodymeniophycidae (Appendix 1). Total genomic DNA (gDNA) from eleven samples was extracted using the commercial ZR Plant/Seed DNA kit (Zymo Research, CA, USA), following the manufacturer's instructions. We amplified *rpoC1* (DNA‐directed RNA polymerase subunit beta') from the gDNA using the manually designed gene‐specific primers described below (see Appendix 2). For the design of the *rpoC1* primers, the degenerate primers were manually designed based on a 50% consensus rule for the most conserved area (e.g., low p‐distance) using both the software BioEdit (Hall, 1999) and the sliding window sequence variation analyses. Polymerase chain reaction (PCR) was conducted using the commercial Titanium® Taq DNA Polymerase kit (Takara Bio USA, Inc., USA), following the manufacturer's instructions. The PCR settings for the initial amplification tests were 96°C for 4 min, and 40 cycles of 94°C for 40 s, 47°C for 40 s, 72°C for 1 min, and 72°C for 10 min. To reduce nonspecific amplification, a Touchdown PCR protocol was carried out as follows: 96°C for 4 min, and 4 cycles of 94°C for 40 s, 52°C for 40 s, 72°C for 1 min, and 2 cycles of 94°C for 40 s, 50°C for 40 s, 72°C for 1 min, and 34 cycles of 94°C for 40 s, 47°C for 40 s, 72°C for 1 min, and 72°C for 10 min. The resulting PCR product was compared against a commercial DNA standard (DM2300 ExcelBand™ 100 bp + 3K DNA Ladder, SMOBiO Technology, Inc., Taiwan) on a 1.5% agarose gel. DNA sequencing was conducted using an ABI3730 DNA Sequencer (Applied Biosystems, Foster, CA) at Mission Biotechnology Company (Taipei, Taiwan).

## 3 RESULTS AND DISCUSSION

**Figure 2:**
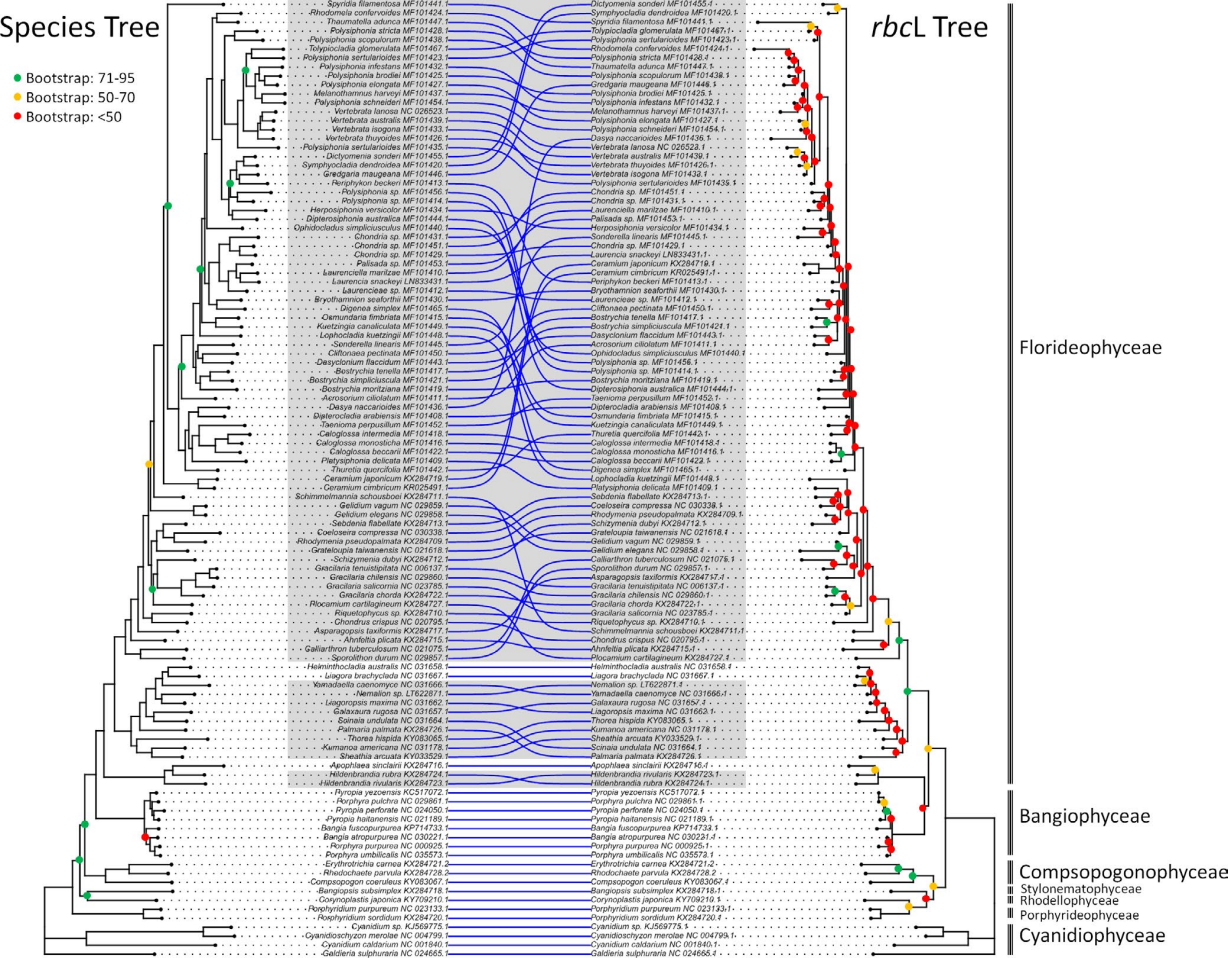
Phylogenies based on the AA alignment concatenated from the 107 core plastid genes (left) and *rbc*L (right). The trees were inferred using RAxML with 100 rapid bootstraps and under the best‐fitting AA models identified by PartitionFinder2. The nodes supported with bootstrap values below 0.95 are color‐coded. Gray shading indicates conflicting nodes between the trees

We developed a bioinformatics strategy to select phylogenetic markers informed by an analysis of 107 published plastid genomes, using these to assemble the AA and NT alignments and the gene trees of 120 single‐copy core plastid gene families. Only 120 protein‐coding genes were retained based on our filtering criteria (i.e., genes were excluded if they were poorly or inconsistently annotated, duplicated, had AA length less than 50, or occurred in less than ~90% of the taxa). We also inferred two trees that represent our best plastid genome‐based estimates of the Rhodophyta phylogeny, one using the AA alignment concatenated from all the plastid genes and the other using the corresponding NT alignment. Overall, the AA plastid genome phylogeny (Figure 2) supports the major interclass relationships observed in published multi‐locus and plastid genome analyses (Cho, Choi, Lam, Kim, & Yoon, 2018; Yang et al., 2016); the corresponding NT phylogeny is largely consistent with the AA phylogeny (nRF = 0.0673; Appendix 3); for example, seven well‐supported monophyletic classes in three subphyla were recovered: one in Cyanidiophytina (Cyanidiophyceae), four in Proteorhodophytina (Compsopogonophyceae, Porphyridiophyceae, Rhodellophyceae, and Stylonematophyceae), and two in Eurhodophytina (Bangiophyceae and Florideophyceae).

Next, we assessed how well each of the plastid genes topologically approximates the plastid genome trees. We ranked the plastid genes by the nRF distance between their trees (i.e., each plastid gene tree) and a target plastid genome tree. In both sets of the nRF rankings of the AA and NT gene trees (Table S2 in Dryad), we found that *psaA* and *psaB* approximate the plastid genome trees better than *rbc*L and *psbA* (i.e., having lower nRF distances to the target trees). A visual comparison of the AA plastid genome tree and the AA *rbc*L gene tree confirms that the *rbc*L gene tree poorly approximates the plastid genome tree (Figure 2). Our findings further support that each of those commonly used plastid markers (i.e., *psaA*, *psaB*, *psbA*, and *rbc*L) alone is not the optimal marker to approximate the red algal phylogeny, consistent with previous observations (e.g., Boo et al., 2016; Lam et al., 2016; Nelson et al., 2015; Verbruggen et al., 2010). Our results also demonstrate that those four popular markers provide limited phylogenetic resolution at the shallow (here, species) levels. This is a known issue with *rbc*L—the most widely employed marker in the red algae (Freshwater, Tudor, O’Shaughnessy, & Wysor, 2010; Yang et al., 2008). In a recent multi‐locus phylogenetic study of the Gelidiales (Boo et al., 2016), *psaA*, *psbA*, and *rbc*L were shown to have peak phylogenetic signals at the deeper levels of the Gelidiales tree rather than at the shallower levels.

Various quantities have been proposed as key criteria for marker gene selection (e.g., Janouškovec et al., 2013; Lei et al., 2012; Yang & Boo, 2006). They include p‐distance, proportion of parsimony informative sites (Pi), and the rates of nonsynonymous substitution (dN) and synonymous substitution (dS). Genes having higher p‐distance, Pi, dN, and/or dS tend to be more suitable for phylogenetic analysis because they harbor more sequence variation, especially when the target clade is an evolutionarily young lineage. Based on the nRF distance rankings alone, it was not apparent how to determine a cutoff to select candidate markers. For instance, in the ranking of the AA trees, about 11 genes have similar nRF distances of ~ 0.2 (Figure 4); also, in this ranking, *gltB* appears to perform better than the other plastid genes. Hence, we examined the p‐distance, Pi, dN, and dS of the plastid genes (Table S2 in Dryad) jointly with the nRF distances to find a clearer cutoff. P‐distance is negatively correlated with the nRF distance between the AA gene trees and AA plastid genome tree (*p* = 2.16 × 10^−7^, Spearman's test; Figure 4); likewise, Pi and dN are negatively correlated with nRF distance (*p* = 1.30 × 10^−6^; not shown). Indeed, p‐distance is positively correlated with dN and Pi (*p* < 2.2 × 10^−16^ for both). However, dS is not correlated with nRF distance (*p* = .10; not shown), probably due to substitution saturation.

**Figure 3:**
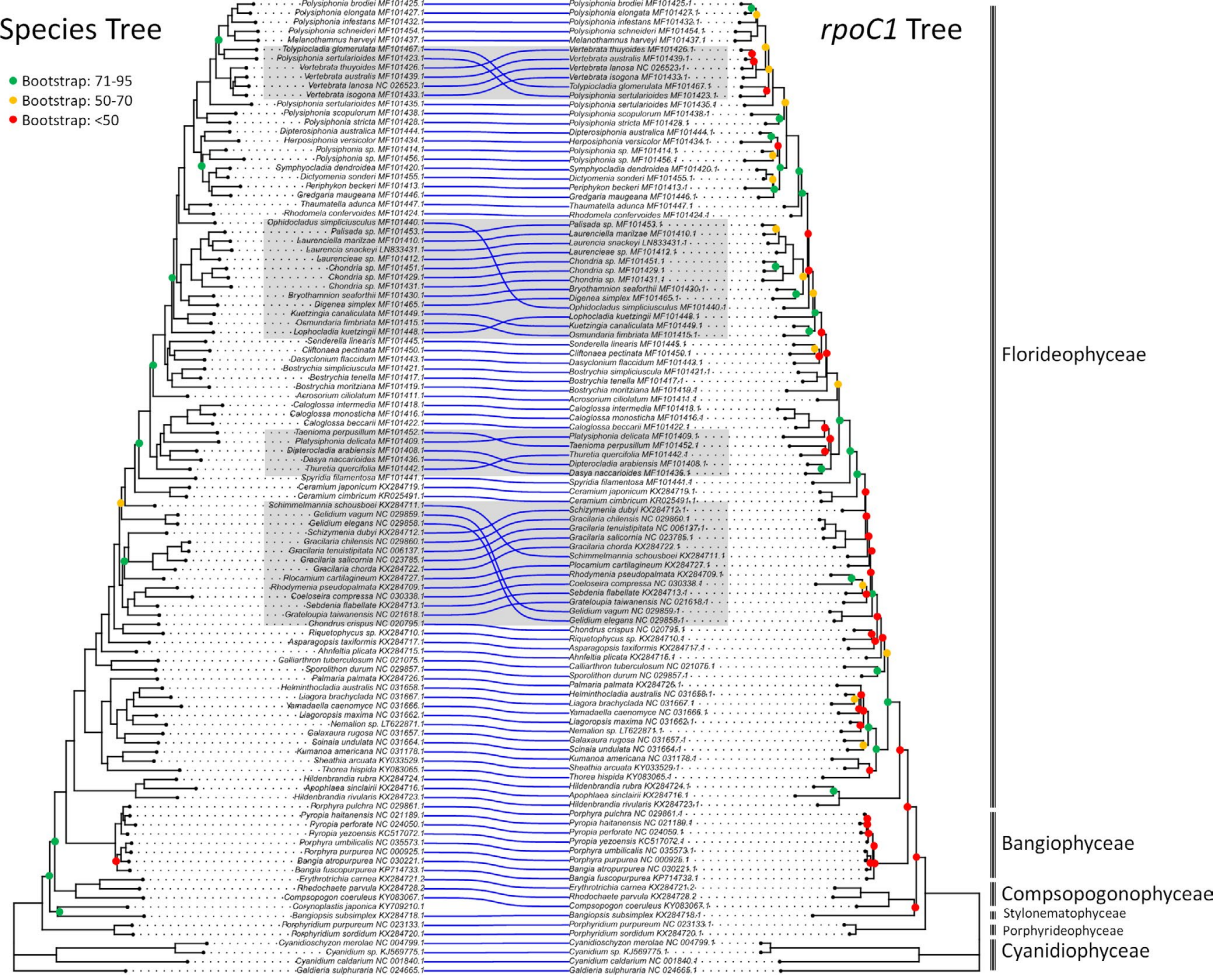
Phylogenies based on the AA alignment concatenated from the 107 core plastid genes (left) and *rpoC1* (right). The trees were inferred using RAxML with 100 rapid bootstraps and under the best‐fitting AA models identified by PartitionFinder2. Nodes supported with bootstrap values below 0.95 are color‐coded. Gray shading indicates conflicting nodes between the trees. *Corynoplastis japonica* was not included in *rpoC1* due to missing coding sequence annotation

**Figure 4:**
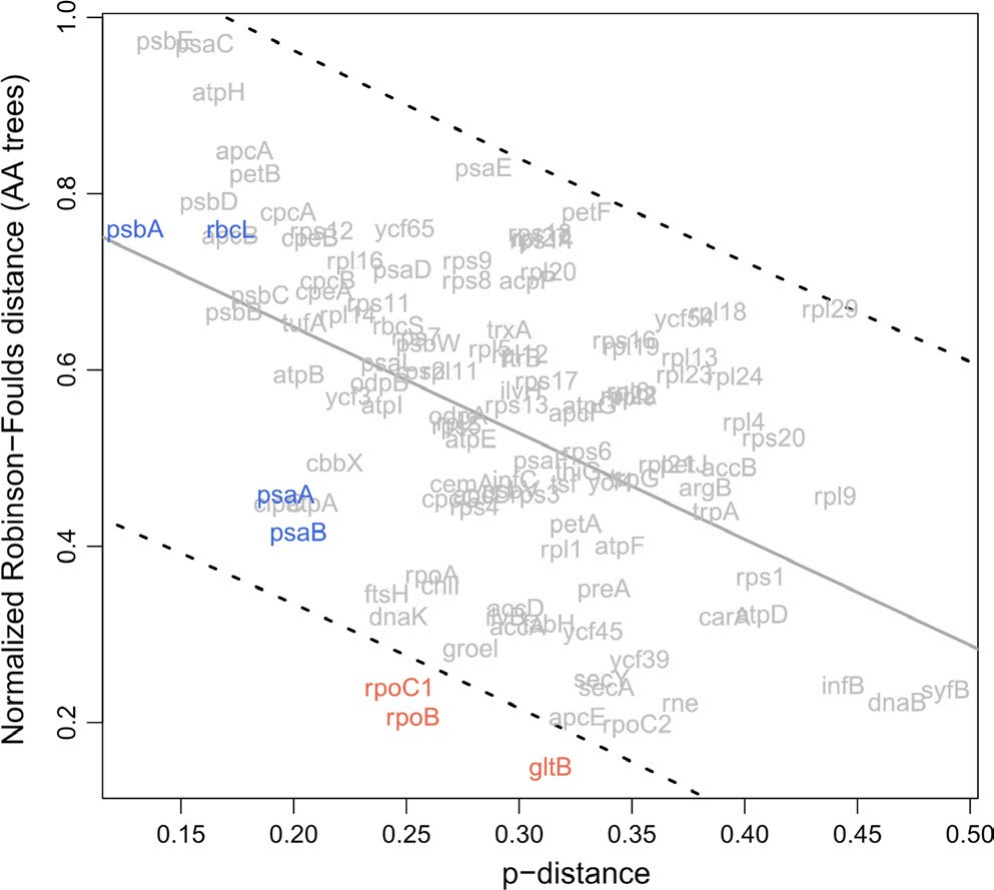
Negative correlation between the normalized Robinson–Foulds (nRF) distance to a target tree and p‐distance across the plastid genes. The nRF distance was calculated based on AA gene trees and a AA plastid genome tree. The dashed lines delineate the 95% prediction interval. Genes that fall below the lower bound of the interval (i.e., low distance and therefore more similar to the target tree) are construed to perform better than other plastid genes having a similar p‐distance. Located inside the interval are the popular plastid markers: *rbc*L, *psbA*, *psaA*, and *psaB* (blue). Below the lower bound of the interval are three genes that are the focus of PCR primer design and testing here: *rpoC1*, *rpoB*, and *gltB* (orange)

When examining the correlations, we noticed that some genes have trees more similar to the target plastid genome trees (i.e., lower nRF distance) than genes with similar levels of sequence divergence (p‐distance) (Figure 4) or similar AA alignment length (Appendix 4). To pinpoint such genes, we performed a linear regression analysis and determined a 95% prediction interval (PI) around the line of best fit (Figure 4). The genes that lie within the PI perform comparably to genes of similar p‐distance. Using the PI as a guide, we found genes that fall below the lower bound of the 95% PI (i.e., having a better nRF distance ranking compared to genes of similar p‐distances or AA alignment length); congruent results were found using NT‐based p‐distances (not shown). In the analysis of the AA data, three genes stood out: *rpoC1*, *rpoB*, and *gltB* (Figure 4), indicating that these outlying genes yield more “accurate” phylogenetic signal (i.e., closer to the target plastid genome tree) than expected based on the amount of sequence information. This approach revealed the same genes even when using dN or Pi instead of p‐distance. In an additional bootstrapping analysis, we took into account uncertainty in tree topology due to sampling errors (i.e., the statistical support of bipartitions). We took 100 bootstrap replicates of a target gene tree and 100 replicates of the plastid genome tree (obtained from the RAxML analysis of the AA MSAs), and randomly drew each with replacement 100 times and then calculated the median nRF distance across the 100 draws. This analysis revealed that the three marker genes still fall outside the 95% PI (Appendix 4), supporting the candidacy of the genes. A visual juxtaposition of the AA plastid genome tree and the AA *rpoC1* gene tree confirms that the *rpoC1* gene tree yields a better approximation of the plastid genome tree (Figure 3) than traditional marker genes, such as *rbc*L (Figure 2).

**Figure 5:**
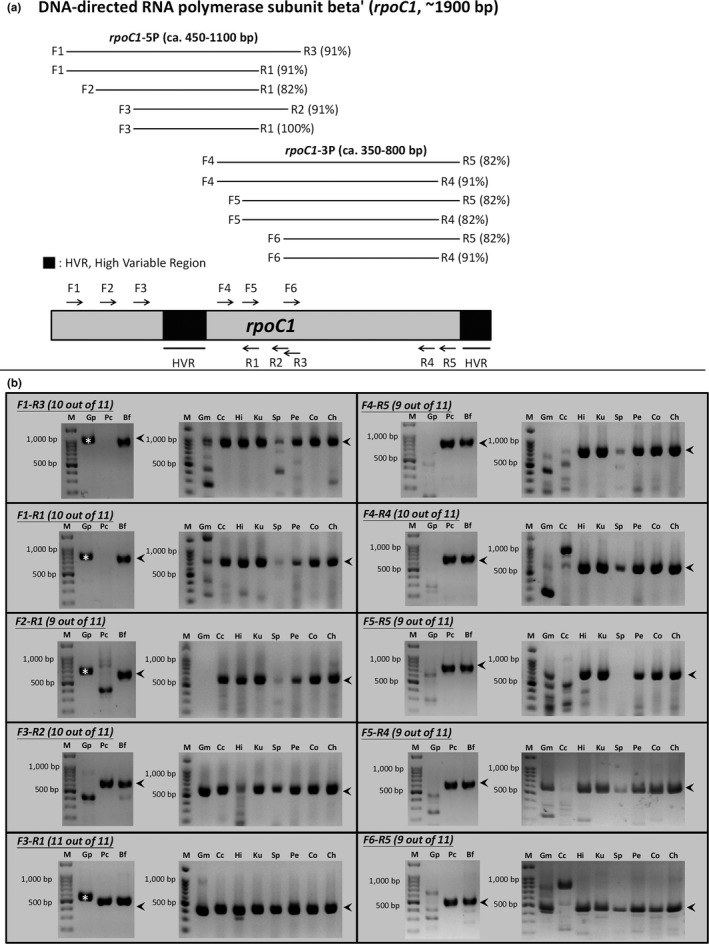
PCR primers designed for *rpoC1* (a) and their amplification efficacy (b) across major taxonomic groups (Cyanidiophyceae: Gm and Gp; Porphyrideophyceae: Pc; Compsopogonophyceae: Cc; Bangiophyceae: Bf; Hildenbrandiophycidae: Hi; Nemalionophycidae: Ku; Corallinophycidae: Sp; and Rhodymeniophycidae: Pe, Co, and Ch). The gene amplification rate was good (82%) or excellent (91% or 100%) for all the primers (shown in parentheses). Two highly variable regions in *rpoC1* exhibit a high level of sequence divergence according to the gene's p‐distance profile. PCR was considered successful if a band of the expected size (indicated by an arrowhead) was observed, even if the band was faint. Unexpected band sizes are nonspecific (or off‐targeted) PCR products. A large amplicon in Gp was observed (marked by asterisks) that is caused by an insertion in the highly variable region confirmed by Sanger sequencing. Abbreviation: Bf, *Bangia fuscopurpurea*; Cc, *Compsopogon caeruleus*; Ch, *Champia* sp.; Co, *Caloglossa ogasawarensis*; Gm, *Galdieria maxima*; Gp, *Galdieria partita*; Hi, *Hildenbrandia* sp.; Ku, *Kumanoa* sp.; M, 100 bp DNA marker; Pc, *Porphyridium cruentum*; Pe, *Peyssonelia* sp.; and Sp, *Sporolithon* sp

Widely employed genetic markers, such as *rbc*L and *psbA*, are amenable to PCR amplification efficiency and Sanger sequencing. Such markers contain regions conserved enough for PCR primer binding (low sequence divergence), as well as a stretch of nucleotides of appropriate length for Sanger sequencing (i.e., 500 to 1,000 bp). Using these criteria, we performed an initial assessment of the potential of the three newly proposed markers for adoption. Among the three markers, *rpoC1* and *rpoB* have relatively low p‐distances and short sequence length, whereas *gltB* is rather long (~4,800 bp) and therefore not ideal as a marker gene (Figure 4; Appendix 4; Table S2 in Dryad). Hence, we decided to focus on *rpoC1* and *rpoB* for PCR primer design and testing. We took a sliding window approach (30 bp) to measure the p‐distance along the NT alignments of *rpoC1* and *rpoB*, finding several regions that seemed suitable for PCR (Appendix 5). Based on the p‐distance profiles, we designed and optimized PCR primers for those two genes and then tested them on 11 red algal specimens (*Galdieria partita*, *Galdieria maxima*, *Porphyridium cruentum*, *Compsopogon caeruleus*, *Bangia fuscopurpurea*, *Hildenbrandia* sp., *Kumanoa* sp., *Sporolithon* sp., *Peyssonelia* sp., *Caloglossa ogasawaraensis*, and *Champia* sp.; Appendix 1), which were selected to represent some of the major lineages of the Rhodophyta. We designed and tested 11 primers for *rpoC1* (five for the 5′ end and six for the 3′ end; Figure 5a; Appendix 2). We successfully amplified *rpoC1* across all the specimens of Florideophyceae, as well as Bangiophyceae (Figure 5b); the amplification success rates were poor in the specimens of the extant descendants of early branching lineages (Cyanidiophyceae, Porphyrideophyceae, and Compsopogonophyceae) (Figure 5b). Based on these PCR results, we suggest two primer pairs, F1‐R3 and F4‐R4, for amplifying *rpoC1*, as they have a high amplification success rate and their overlapping PCR products span most of *rpoC1* (validated by Sanger sequence data, which were deposited in NCBI GenBank; Appendix 1). We also tried testing F1‐R4 and F1‐R5 a few times, but had a low success rate with F1‐R4 (25%; only in *Compsopogoncaeruleus* and *Hildenbrandia* sp.; data not shown) and no amplification for the rest of the specimens. Moreover, we could not achieve the same level and consistency of success with *rpoB* even after several attempts at primer design and testing, probably because this gene is more divergent (Appendix 5), longer (3,386 bp), and lower in GC content (32.64%) than *rpoC1*.

Many attractive phylogenetic markers may not be suitable for PCR primer design for various reasons, hampering their uptake by the research community. Furthermore, it is known that in amplicon‐based eDNA metabarcoding studies, estimates of relative abundance are skewed, and so our estimates of community species diversity may be poorer than they could be (e.g., Wilcox et al., 2018). However, there exist alternative technologies that could enable researchers to sequence such markers without needing to go through the laborious process of PCR primer development. For example, one can utilize the plastid markers proposed using our in silico methodology in an approach that leverages both HTS and probe‐based target hybridization (e.g., Shokralla et al., 2016; Weitemier et al., 2014). Probes (or baits) can be designed to bind to the plastid markers (“targets”), and the bait‐target complexes would be pulled down or enriched (for example, using magnetic beads that bind to biotinylated baits) while nontarget nucleic acids are washed away. This method effectively enhances the ratio of target to nontarget nucleic acids, and the resulting target‐enriched pool of nucleic acids can then be subjected to HTS (e.g., Mariac et al., 2018). This would exploit the scalability of HTS to facilitate eDNA metabarcoding studies of the red algae that have thus far been infeasible (e.g., due to PCR amplification failure). Moreover, if the target genes are too long for short‐read HTS technologies by Illumina Inc., long‐read sequencing technologies, such as the MinION by Oxford Nanopore Technologies Ltd., provide a promising alternative approach. The handheld, affordable, and field‐deployable MinION boasts long sequencing read lengths of thousands to millions of base pairs long (e.g., Krehenwinkel et al., 2019). This powerful feature enables the sequencing of entire genes without the need to correct for assembly errors (i.e., chimeric sequences) (see Saunders & Moore, 2013). The MinION has been criticized for its high base‐calling error rate, but it is anticipated that it will be improved in upcoming technological updates. Evaluating the utility of a target hybridization‐based HTS eDNA metabarcoding approach, coupled with nanopore sequencing and with phylogenomic approaches such as ours, could be a productive avenue for future research.

Taxon sampling is an important consideration when choosing an appropriate phylogenetic marker. Here, we examined all the plastid genomes available to us at the beginning of the study (Dec. 2017). Nearly half of the taxa (53 of 107; 51%) were sampled from the most species‐rich family Rhodomelaceae (Ceramiales), which encompasses roughly 15% of the recognized species diversity of the Rhodophyta (AlgaeBase; Guiry & Guiry, 2019). We intended to search for phylogenetic markers that would allow us to recover shallow relationships (e.g., species‐ or population‐level) for phylogenetic community analysis, because we were not attempting to investigate the deep relationships of the red algal tree of life. Hence, our sampling is biased toward Rhodomelaceae, and therefore, the marker rankings and the proposed *rpoC1* marker may be more pertinent to this family. We anticipate to identify and test candidate markers that are more specific for focal clades (orders, e.g., Corallinales, Gigartinales, and Rhodymeniales; or families within Ceramiales, e.g., Ceramiaceae and Delesseriaceae) as their plastid genomes become available. Moreover, we hope to maintain these marker rankings alongside with curated sequences as a resource for the phycological community, beginning with *rpoC1*. Presently, we are conducting broader testing of the *rpoC1* primers on more specimens across more diverse red algal lineages.

## 4 CONCLUSIONS

Much remains to be discovered about the processes shaping the biodiversity and community assembly of the red algae. HTS‐based eDNA metabarcoding utilizing phylogenetic community analysis based on carefully selected markers will help to elucidate those processes. There is a scarcity of tools and resources (robust phylogenetic markers, well‐tested PCR primers, optimized wet‐lab protocols, and high‐quality reference sequence databases) for the eDNA metabarcoding of the red algae. By leveraging the genomic resource contributed cumulatively by the phycological community, we have taken the first step toward the long‐term goal of building additional tools and resources. Finally, expansion of similar efforts to mine mitochondrial and nuclear genomes and periodic re‐evaluation of plastid genomes, as more and more data become available, may help to augment the molecular toolbox to investigate the phylogenetic community ecology of the red algae.

## CONFLICT OF INTEREST

None declared.

## AUTHOR CONTRIBUTION

SHZ and SLL conceived the project. SHZ analyzed the data. CCS and SLL conducted the experiment. SHZ and SLL prepared the manuscript and contributed substantially to revisions.

## Open research badges

This article has earned Open Data, Open Materials and Preregistered Research Design badges. Data, materials and the preregistered design and analysis plan are available at Dryad (https://doi.org/10.5061/dryad.4qrfj6q62) and GitHub (https://github.com/szhan/rhododb).

## Data Availability

DNA sequences: NCBI GenBank accession numbers MN538998‐MN539008for *rpoC1*, MH835528, MH835647, MH835676, MN431657, MN539012‐MN539016, and MN540180 for *rbc*L, and MN431657, MN539009‐MN539011, and MN540181for *psbA*. Online supplementary files: Dryad (https://doi.org/10.5061/dryad.4qrfj6q62) and GitHub (https://github.com/szhan/rhododb).
