# Supplementary material for: Reappraising plastid markers of the red algae for phylogenetic community ecology in the genomic era
Source: Ecol Evol. 2020 Jan 11;10(3):1299–310. doi: 10.1002/ece3.5984 (PMC7029088; doi:10.1002/ece3.5984)
Supplement: Supplementary file 1 [file ECE3-10-1299-s001.docx]

**Appendix 1**. Taxa used in the PCR experiments, their collection information, and NCBI GenBank accessions.

| **Taxon** | **Class/Subclass** | **Collection Information** | **Accession** | | |
| --- | --- | --- | --- | --- | --- |
|  |  |  | ***rpoC1*** | ***rbc*L** | ***psbA*** |
| *Galdieria partita* Sentsova | Cyanidiophyceae/- | Strain THAL043, DaYouKen, Yangmingshan National Park, Taiwan | MN538998 | MN540180 | MN540181 |
| *Galdieria maxima* Sentsova | Cyanidiophyceae/- | Strain THAL066, GenZiPing, Yangmingshan National Park, Taiwan | MN538999 | MN431657 | MN431657 |
| *Porphyridium cruentum* (Gray) Nägeli | Porphyridiaceae/- | Strain UTEX161, wet shaded tuff, Basel, Switzerland | MN539000 | MN539012 | MN539009 |
| *Compsopogon caeruleus* (Balbis ex Agardh) Montagne | Compsopogonophyceae/- | Voucher THU.368, "GouLaoBan" Pet Shop, Section 1, Jieshou Road, Bade District, Taoyuan, Taiwan | MN539001 | MH835676 | - |
| *Bangia fuscopurpurea* (Dillwyn) Lyngbye | Bangiophyceae/- | Voucher YASP116, Yongxin, Taoyuan Algal Reef, Taoyuan, Taiwan | MN539002 | MN539013 | MN539010 |
| *Hildenbrandia* sp. | Florideophyceae/ Hildenbrandiophycidae | Voucher BYSP051, Baiyu, Taoyuan Algal Reef, Taoyuan, Taiwan | MN539003 | MN539014 | - |
| *Kumanoa* sp. | Florideophyceae/ Nemaliophycidae | Voucher THU.571, A spring at Kenting National Park, Hengchun, Taiwan | MN539004 | MH835528 | - |
| *Sporolithon* sp. | Florideophyceae/ Corallinophycidae | Voucher DTG1SP025, Datan, Taoyuan Algal Reef, Taoyuan, Taiwan | MN539005 | - | MN539011 |
| *Peyssonnelia* sp. | Florideophyceae/ Rhodymeniophycidae | Voucher YASP034, Yongxin, Taoyuan Algal Reef, Taoyuan, Taiwan | MN539006 | MN539015 | - |
| *Caloglossa ogasawaraensis* Okamura | Florideophyceae/ Rhodymeniophycidae | Voucher THU.423, ChuanZiTou Bridge, Dahu Road, Yuanshan Township, Yilan, Taiwan | MN539007 | MH835647 | - |
| *Champia* sp. | Florideophyceae/ Rhodymeniophycidae | Datan, Taoyuan Algal Reef, Taoyuan, Taiwan | MN539008 | MN539016 | - |
