# Supplementary material for: Reappraising plastid markers of the red algae for phylogenetic community ecology in the genomic era
Source: Ecol Evol. 2020 Jan 11;10(3):1299–310. doi: 10.1002/ece3.5984 (PMC7029088; doi:10.1002/ece3.5984)
Supplement: Supplementary file 2 [file ECE3-10-1299-s002.docx]

**Appendix 2**. *rpoC1* primers.

| **Designation** | **Sequence** | **Direction** |
| --- | --- | --- |
| F1 | 5’-GAAAGAAYWTTRCCWAATGG-3’ | Forward |
| F2 | 5’-GAYTGGGARTGTCAYTGTGG-3’ | Forward |
| F3 | 5’-ACTCATGTTTGGTAYYTAAAAGG-3’ | Forward |
| F4 | 5’-GGATGRTWTTTTYWGTWATWCC-3’ | Forward |
| F5 | 5’-GATGGWGGWMGWTTTGCWACWGC-3’ | Forward |
| F6 | 5’-GATATTATWGAAGGWAARCARGG-3’ | Forward |
| R1 | 5’-GCWGTWGCAAAWCKWCCWCCATC-3’ | Reverse |
| R2 | 5’-CCATTATCCATTARWGMATCWACWGC-3’ | Reverse |
| R3 | 5’-CCYTGYTTWCCTTCWATAATATC-3’ | Reverse |
| R4 | 5’-GCCATTTGATCWCCATCRAAATC-3’ | Reverse |
| R5 | 5’-CCATRTCTTGRCTWGGCAT-3’ | Reverse |
