# Supplementary material for: Reappraising plastid markers of the red algae for phylogenetic community ecology in the genomic era
Source: Ecol Evol. 2020 Jan 11;10(3):1299–310. doi: 10.1002/ece3.5984 (PMC7029088; doi:10.1002/ece3.5984)
Supplement: Supplementary file 3 [file ECE3-10-1299-s003.docx]

**Appendix 3.** A maximum-likelihood phylogeny based on the NT alignment concatenated from the 107 core plastid genes. Bootstrap proportion values based on 100 replicates of maximum likelihood analyses were shown beside the node. Scale bar indicates substitutions per site.
