# Supplementary material for: Reappraising plastid markers of the red algae for phylogenetic community ecology in the genomic era
Source: Ecol Evol. 2020 Jan 11;10(3):1299–310. doi: 10.1002/ece3.5984 (PMC7029088; doi:10.1002/ece3.5984)
Supplement: Supplementary file 4 [file ECE3-10-1299-s004.docx]

**Appendix 4.** Negative correlation between the median bootstrap normalized Robinson-Foulds (nRF) distance and p-distance (a) and between the nRF distance and the amino acid alignment length (b). The median nRF distance in (a) was estimated from 100 pairwise comparisons of the bootstrap replicates of an individual gene tree and the target plastid genome tree; the nRF distance in (b) was calculated based on a single tree, as in **Figure 4**. The dashed lines delineate the 95% prediction interval. The popular plastid markers (*psaA*, *psaB*, *psbA*, and *rbc*L) are shown in blue, while the newly proposed three markers (*gltB*, *rpoB*, and *rpoC1*) are shown in orange.
