# Supplementary material for: Reappraising plastid markers of the red algae for phylogenetic community ecology in the genomic era
Source: Ecol Evol. 2020 Jan 11;10(3):1299–310. doi: 10.1002/ece3.5984 (PMC7029088; doi:10.1002/ece3.5984)
Supplement: Supplementary file 5 [file ECE3-10-1299-s005.docx]

**Appendix 5**. P-distance profile of *rpoC1* (a) and *rpoB* (b). P-distance was measured over the NT alignment by sliding a window of 30 bases. For a given window, we calculated the median pairwise p-distance. At p-distance of ~0.2, the regions should be conserved enough for PCR primer design.
